# The influence of amaranth (*Amaranthus hypochondriacus*) dietary nitrates on the aerobic capacity of physically active young persons

**DOI:** 10.1186/s12970-020-00366-5

**Published:** 2020-07-13

**Authors:** Tomas Liubertas, Ramutis Kairaitis, Loreta Stasiule, Sandrija Capkauskiene, Arvydas Stasiulis, Pranas Viskelis, Jonas Viškelis, Dalia Urbonaviciene

**Affiliations:** 1 Department of Coaching Science, Lithuanian Sports University, 44221 Kaunas, Lithuania; 2 Department of Applied Biology and Rehabilitation, Lithuanian Sports University, 44221 Kaunas, Lithuania; 3 Institute of Horticulture, Lithuanian Research Centre for Agriculture and Forestry, 54333 Babtai, Lithuania

**Keywords:** Aerobic capacity, Dietary nitrates, Amaranth, Cycling, Young persons

## Abstract

**Background:**

Recent evidence indicates that elevating plasma nitrites through dietary nitrates (NO_3_^−^) supplementation is associated with enhanced muscle efficiency, fatigue resistance and performance. Beetroot (in various forms) is the dominant source of dietary NO_3_^−^ primarily due to its vast availability and the simple form of preparation suitable for final consumption. After a few years of research and experimentation, our scientific team identified alternative source rich with dietary NO_3_^−^ as possible nitric oxide precursor, amaranth (*Amaranthus hypochondriacus*) with a standardized concentration 9–11% of NO_3_^−^. This study aimed to evaluate the effect of single-dose (±400 mg of dietary NO_3_^−^) and long-term (6 days) supplementation of amaranth concentrate derived dietary NO_3_^−^ on aerobic capacity in physically active young people.

**Methods:**

We conducted a randomized, double-blind, placebo-controlled human study. Thirteen healthy and physically active young male participants were randomized into experimental and placebo groups. The aerobic capacity was tested during increasing cycling exercise (ICE) with pulmonary gas exchange recording and analysis.

**Results:**

The peak power of the ICE, the maximum oxygen consumption and the first ventilatory threshold were significantly increased after long-term consumption of dietary amaranth (from 4.44 ± 0.50 to 4.55 ± 0.43 W/kg; from 37.7 ± 2.7 to 41.2 ± 5.4 mL/kg/min and from 178.6 ± 30.3 to 188.6 ± 35.2 W, *p* < 0.05; respectively) in experimental group.

**Conclusions:**

Long-term (6 days) use of dietary NO_3_^−^ from amaranth may improve the aerobic capacity during ICE in young physically active male persons. It can be recommended as the nutritional supplement during last week of preparation for competition in endurance events.

## Introduction

Green leafy vegetables and roots are the main source of dietary nitrates (NO_3_^−^) [1–3]. NO_3_^−^ is a naturally occurring compound as well as an approved food additive [1, 2]. A number of studies have already confirmed the benefits of dietary nitrates to human health [4]: their consumption reduces blood pressure, suppresses platelet aggregation, protects against ischemic diseases, and improves endothelial function [1]. Nitric oxide and nitrites, both NO_3_^−^ products, affect vasodilatation by increasing blood flow [5], thus increasing the oxygen uptake and oxidative processes in the working muscles [6]. Additionally, nitrates show to increase the bioavailability of blood plasma, which is important for the exogenous pathway of nitrates-nitrite-NO and acts as a regulator of hypoxic signals and NO-induced vasodilatation [7].

The effects of nitrate/nitrite/NO on the muscle circulatory system and mitochondrial and contractile efficacy [8, 9] may increase muscle blood flow circulation and improve the metabolic response to physical activity [10]. The evidence supports that even the concentration of plasma nitrites is an independent factor of physical performance [5, 11]. Nevertheless, studies on the effects of nitrates on work capacity indicators are highly controversial so far.

Studies have shown nitrates to have a positive effect on work efficiency and oxygen expenditure [10, 12–17], but other studies have not found visible and conclusive changes in given performance [14, 18–22].

A large number of researchers found that 300–500 mg of beetroot nitrates have a single and long-lasting positive effect on the aerobic performance of physically active individuals [10, 12, 16–19, 23]. Recently became popular and actively researched beetroot (in various forms) is the dominant source of dietary NO_3_^−^, primarily due to its vast availability and the simple form of preparation suitable for final consumption. Remarkably, limited studies have evaluated NO_3_ rich leafy vegetables and, more specifically, amaranth on exercise performance. Importantly, amaranth is not only rich in NO_3_, potassium (> 10% by weight) and antioxidant polyphenols (e.g. amaranthine), but also devoid of sugar and oxalates. It has recently been reported that red spinach extract as a nutritional supplement can elicit an ergogenic response by delaying the ventilatory threshold during graded treadmill exercise testing [24]. After a few years of research and experimentation, our scientific team identified alternative source rich in dietary NO_3_^−^ as possible and alternative nitric oxide precursor, amaranth (*Amaranthus hypochondriacus*) with a standardized concentration 9–11% of NO_3_^−^. Since NO_3_^−^ supplementation increases plasma NO_2_^−^, this intervention may therefore have the potential to improve muscle blood flow [5], thus increasing the oxygen uptake and oxidative processes in the working muscles [6] and exercise tolerance. Thus, based on research data available we formulated the hypothesis that 400 mg of dietary NO_3_^−^ from amaranth (dietary amaranth) will increase the aerobic capacity of physically active young people.

In this study we aimed to evaluate the effect of single and long-term doses of dietary amaranth on the aerobic capacity of physically active young persons.

## Materials and methods

### Participants

The study recruited 13 volunteering graduate students (all males) from Lithuanian Sports University. Every participant was informed about the research objectives and methods and signed an Informed Consent form for participation. The study was conducted in accordance with the Declaration of Helsinki, and the protocol was approved by the Kaunas Regional Ethics Committee, Nr. BE-2-11, 21 March 2017. The anthropometric data and age of the participants are presented in Table 1.

**Table 1:** Characteristics of study participants

Variable	Placebo group^1^	Experimental group^2^	*p*-Value
Age (years)	21.9 ± 1.9	21.3 ± 0.8	0.940
Height (cm)	182.1 ± 5.9	180.5 ± 8.3	0.829
Body weight (kg)	88.1 ± 12.5	84.3 ± 23.7	0.668
Body mass index (BMI)	26.5 ± 2.8	25.6 ± 5.8	0.568
Relative fat mass (%)	13.1 ± 3.8	18.9 ± 5.6	0.063

Values are reported as the mean ± standard deviation (SD). ^1^*n* = 6; ^2^*n* = 7

### Measurements

#### Anthropometry

Electronic weighing scales (Body Composition Analyzer TBF-300, Tanita, Japan). were used to measure the weight and relative fat mass of the participants. Height was measured using a stadiometer (Leicester height measure, UK).

#### Increasing cycling exercise (ICE)

For the assessment of aerobic capacity, an ICE was performed on a cycle ergometer (Ergoline-800, Denmark). The seat and handlebar positions on the cycle ergometer were adjusted for each subject prior to the initial exercise test and maintained in that position for the subsequent exercise tests. Prior to the ICE, a 5-min warm-up was performed. The ICE consisted of 3 min of cycling at 40 W, then the ramp protocol was applied, and the workload was continuously increased by 30 W per min. The cadence was 70 rpm. The participants were encouraged to exercise until voluntary fatigue, and the test was stopped when the participant was not able to maintain a cadence above 65 rpm.

#### Pulmonary gas exchange recording and analysis

The subjects breathed through a face mask, and pulmonary gas exchange parameters were measured breath-by-breath using a wireless, portable spirometric system “Oxycon Mobile” (Viasys Healthcare; California, USA). Prior to each exercise session, the spirometric system was calibrated according to the recommendations of manufacturers. The maximum value of oxygen uptake (VO_2_) over the 20 s of cycling was referred to as VO_2_ max, and the first and second ventilatory thresholds (VT1 and VT2) were determined from the data of the incremental cycling exercise. The determination was based on the analysis of the relationship between ventilatory equivalents of oxygen or carbon dioxide and cycling power. The VT1 was identified as the first point at which the ventilatory equivalent for O_2_ increased without a concurrent increase in the ventilatory equivalent for CO_2_. The VT2 was identified as the point of constant increase of ventilatory equivalent of CO_2_. A least squares method was used to fit two lines representing ventilatory equivalents versus load plots. The intersection point of the two regression lines was assigned to VT1 and VT2. Heart rate (HR) was recorded continuously with a wireless Polar monitoring system (Polar, Finland).

#### Biochemical analysis of blood

Blood samples for the measurement of blood lactate concentration [La] (Lactate Pro2, Japan) were taken from fingertips at the end of the 5th min of recovery after the ICE.

### Protocol

**Fig. 1:**
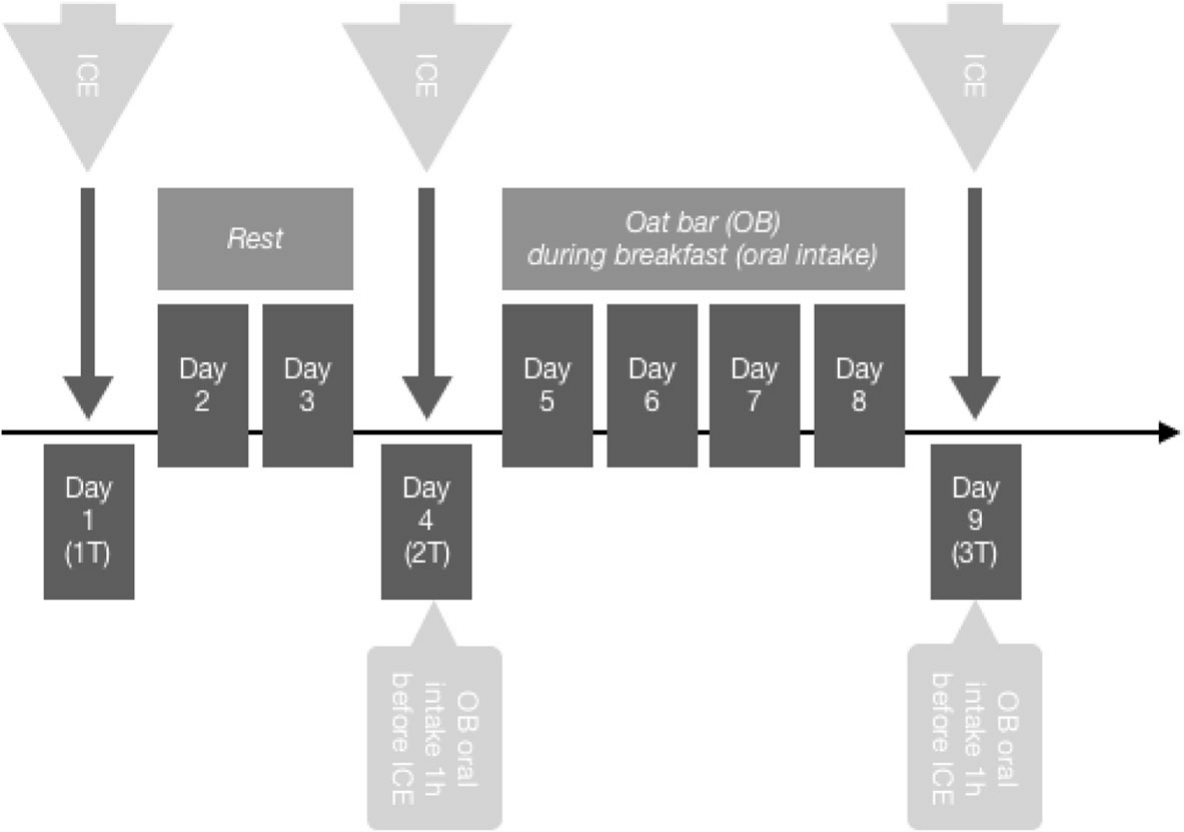
Study protocol. Note: ICE – increasing cycling exercise test; OB - handmade oat bar; 1–9 –days of study. (1 T) - test Nr. 1 executed at day 1 of the study; (2 T) - test Nr. 2 executed at day 4 of the study; (3 T) - test Nr. 3 executed at day 9 of the study

A randomized double-blinded design was used in this study. During the first visit, the participants had their anthropometric measurements taken and performed the ICE (1 T) (Fig. 1).

After 2 days of rest, participants repeated the ICE (2 T) 1 h after consumption of supplement with (experimental group) or without (placebo group) *Amaranthus hypochondriacus*. Then, participants in both groups consumed supplement for 4 days during breakfast and, on the next day, performed a third ICE (3 T) after consuming supplements 1 h before the test.

The experimental group consumed a hand-made oat bar supplement (OB) 60 g total weight - made of oats, honey, vanilla, containing 4 g standardized *Amaranthus hypochondriacus* concentrate (9–11% equivalent to ±400 mg of active ingredient (NO_3_^−^). The placebo group consumed visually and flavory identical oat bar (OB) - 60 g, containing oats, honey and vanilla (excluding active ingredient - ie. *Amaranthus hypochondriacus* concentrate). Participants were asked not to change their nutritional habits during the period of study.

### Statistical analysis

The statistical analysis was carried out with SPSS (Statistical Package for Sοcial Sciences, version 19.0) and Micrοsοft Οffice Excel 2007. The normal distribution of variables was checked using the Kolmogorov-Smirnov test. Non-parametric data analysis methods were used to assess the effect of dietary amaranth on aerobic capacity. The significance of the difference between the independent samples was evaluated using the Mann-Whitney test. The difference between dependent samples was assessed by the Wilcoxon test. Statistical significance was accepted when *p* < 0.05. All data are reported as the mean ± standard deviation (SD).

## Results and discussion

### Results

**Fig. 2:**
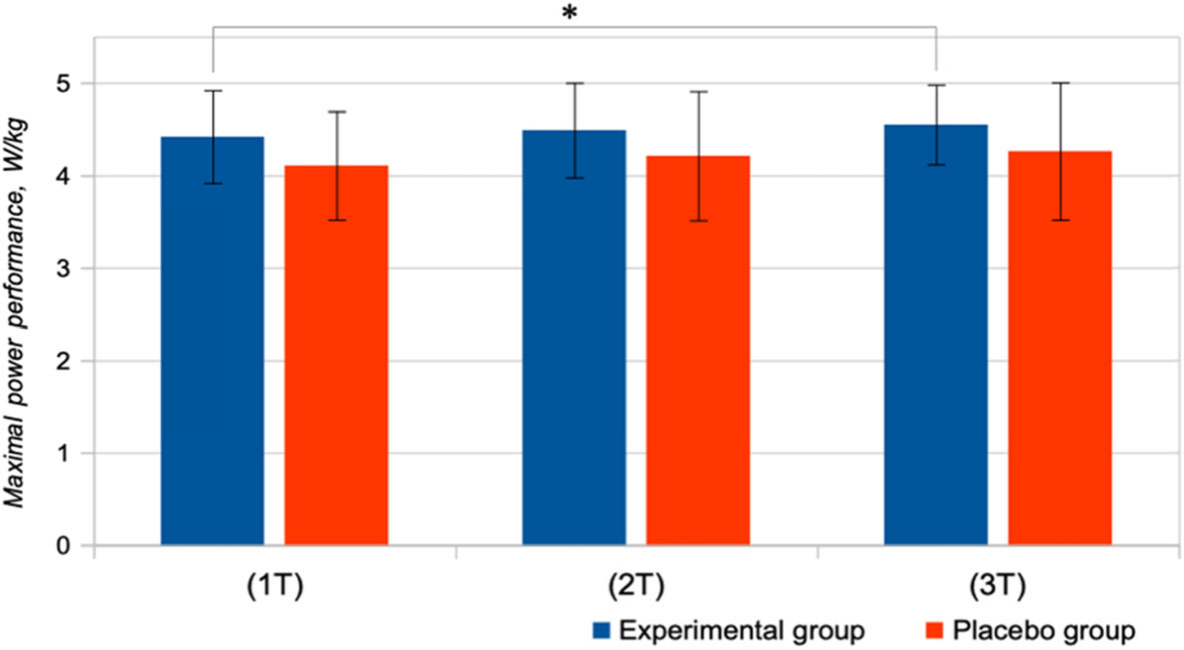
Peak power of increasing cycling exercise (ICE) following single-dose (2 T) and long-term (3 T) doses of supplements in the experimental and placebo groups. * *p* < 0.05 - statistically significant difference compared to the 1 T

**Fig. 3:**
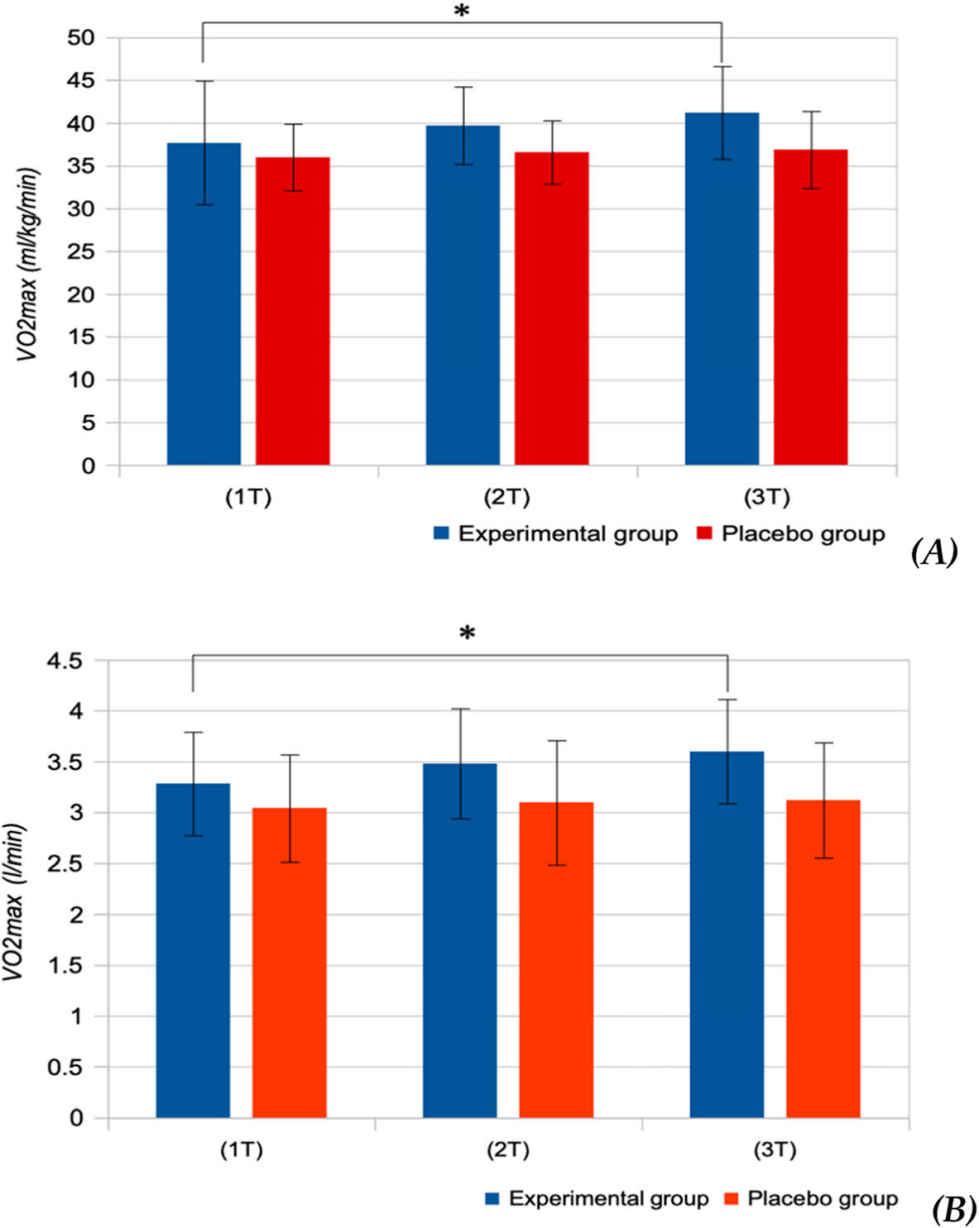
Maximal oxygen uptake (VO2max) following single-dose (2 T) and long-term (3 T) doses of supplements in the experimental and placebo groups. * p < 0.05 - statistically significant difference compared to the 1 T

**Fig. 4:**
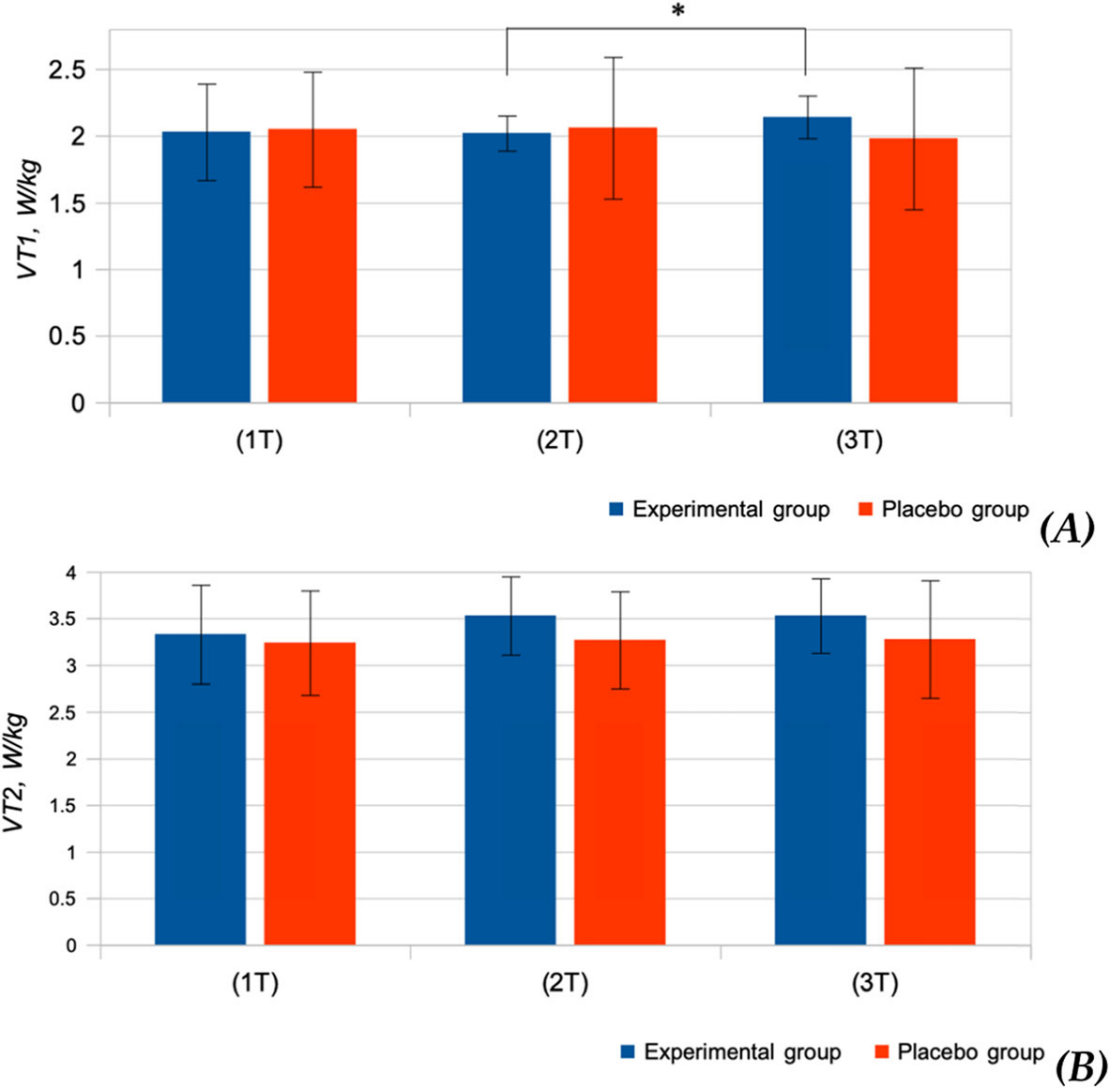
First ventilatory threshold (VT1) and second ventilatory threshold (VT2) following single-dose (2 T) and long-term (3 T) doses of supplements in the experimental and placebo groups. * p < 0.05- statistically significant difference between the 2 T and 3 T

No significant changes of the parameters of aerobic capacity were observed in Placebo group (Figs. 2, 3, 4). Peak power of ICE has increased significantly in the experimental group, from 4.42 ± 0.50 W/kg during the first testing to 4.55 ± 0.43 W/kg during the third testing (*P* = 0.043; Fig. 2). The single dose of supplements did not have any significant effect on the VO_2_max in the experimental group. After long-term use of dietary amaranth, absolute and relative values of VO_2_max demonstrated a significant increase in the experimental group (from 3.282 ± 0.51 l/min and 37.7 ± 2.7 mL/kg/min during the first test to 3.599 ± 0.51 l/min (*p* = 0.028) and 41.2 ± 5.4 mL/kg/min (*p* = 0.043) during the third test, respectively (Fig. 3).

VT1 did not change after a single dose of dietary amaranth, but a significant difference was observed in the experimental group after long-term supplementation (third testing). After a single dose dietary amaranth VT1 was 2.02 ± 0.13 W/kg and after long-term use of dietary amaranth the value of VT1 increased significantly to 2.14 ± 0.16 W/kg (*p* = 0.028; Fig. 4a). The VT2 did not change significantly in any of the groups (*p* > 0.05, Fig. 4b).

Other physiological variables measured during ICE did not change significantly in any group of participants (Table 2).

**Table 2:** Maximal physiological responses during increasing cycling exercise following single-dose (2 T) and long-term (3 T) doses of supplements in the experimental and placebo groups (HR - heart rate; RER - respiratory exchange ratio; [La] - blood lactate concentration)

Indexes	Experimental group		
	1 T	2 T	3 T
HR, beats/min	186.1 ± 8.2	183.8 ± 9.1	184.2 ± 7.4
RER	1.18 ± 0.07	1.16 ± 0.04	1.24 ± 0.06
[La] at 5th min after ICE, mmol/l	14.3 ± 2.9	15.2 ± 4.8	12.4 ± 1.6
Indexes	Placebo group		
	1 T	2 T	3 T
HR, beats/min	189.2 ± 5.7	188.2 ± 8.1	187.0 ± 7.6
RER	1.20 ± 0.07	1.16 ± 0.04	1.22 ± 0.07
[La] at 5th min after ICE, mmol/l	13.0 ± 2.1	13.6 ± 4.6	13.9 ± 4.2

Key: Values are expressed as mean ± SD

### Discussion

In the present study we analyzed the effect of single dose (equivalent to ±400 mg) and long term consumption of dietary nitrates obtained from amaranth (*Amaranthus hypochondriacus)* on aerobic capacity of healthy physically active young people. Our principal findings are that the VO_2_max, VT1 and peak power measured during gradually increasing cycling exercise increased significantly only after 6 days of supplementation, but demonstrated no significant effect after a single dose.

Our data coincides with other studies of a similar nature carried out by other authors (Table 3). The researchers cited used beetroot juice with varying amounts of NO_3_. The most popular use of the fixed duration of the dietary NO_3_ is 4–6 days, and the amount varies from 5 mmol up to 9 mmol. Positive and significant effect for VO_2_max was found by Bailey [10, 12], Lansley [16], Vonhatalo [18], and Wylie [11]. Their studies involved healthy, physically active people and tested physical performance. Vanhatalo [18] explored the long-term effects of nitrates with 5 and 15 days of 500 ml of 5.2 mmol nitrate from beetroot juice and found that the 15-day period significantly improved the maximum aerobic power (Table 3). Most previous studies have used beetroot juice as a source of dietary NO3–, but only a few have investigated the effect of other sources. In one study, the immediate ergogenic effect (delaying the ventilatory threshold) of red spinach extract was demonstrated [24]. The authors reported that after acute ingestion of 1000 mg of red spinach extract, VO2 at the ventilatory threshold was significantly higher, although no significant changes were seen in the time-to-exhaustion or maximal aerobic power. By contrast, in our study, significant changes were observed after 6 days of dietary amaranth consumption. Our dose of NO3– was lower (400 mg) than that in the report by More et al. [25], and this could be why a single dose had no effect.

**Table 3:** Effects of single and long-term use of nitrates on the indicators of work capacity of healthy and physically active persons

References	Participants	Supplemen-tation	Concentration of nitrates mmol/day	Duration of consumption	Protocol	Findings
[12]	Healthy, physically active people, *n* = 8; placebo (P) and experimental groups (E)	500 ml beetroot juice /day	~ 340 mg or 11 mmol	6 days	Time trial at 70% between the ventilation threshold and VO_2_max	The increase in pulmonary O_2_ uptake following the onset of moderate exercise was reduced by 19% in the beetroot juice condition. During severe exercise, the O_2_ uptake slow component was reduced, and the time-to-exhaustion was extended. Trial duration: E: 675 ± 203 s P: 583 ± 145 s (*p* < 0,05)
[19]	Healthy people, *n* = 9;	NaNO_3_	0.033 mmol NaNO_3_ kg/ body WT three times daily	Randomized and double-blinded; the washout period between the two trials was at least 7 days	Maximal combined arm and leg exercise tests.	Dietary nitrate reduced VO_2_max from 3.72+/−0.33 to 3.62+/− 0.31 L/min, *p* < 0.05. Despite the reduction in VO_2_max the time-to-exhaustion trended towards an increase after nitrate supplementation (524+/− 31 vs 563+/− 30 s, *p* = 0.13).
[10]	Healthy, physically active people; *n* = 7	500 ml beetroot juice /day	300 mg or ~ 5,1 mmol	6 days	Leg extension until fatigue at 30% of MVC	20% reduced O_2_ cost of exercise following dietary NO_3_- supplementation appears to be due to a reduced ATP cost of muscle force production. Trial duration: E: 734 ± 109 s P: 586 ± 80 s (*p* < 0,05)
[18]	Healthy, physically active people; *n* = 9	500 ml beetroot juice /day	300 mg or ~ 5,1 mmol	A. 2,5 h before B. 5 days C. 15 days	Increasing exercise test	**A**.E: 325 ± 71 Wmax P: 322 ± 68 Wmax **B**. E:328 ± 68 Wmax P: 323 ± 67 Wmax **C**. E:331 ± 68 Wmax P: 323 ± 68 Wmax After 15 days maximal power was increased by 4%
[16]	Healthy, physically active people; *n* = 9	500 ml of beetroot juice (BR)	380 mg or ~ 6,1 mmol	6 days	Subjects completed treadmill exercise tests and knee-extension exercise tests for estimation of Q_max_.	The O_2_ cost of walking, moderate-intensity running, and severe-intensity running was reduced by BR; time-to-exhaustion during severe-intensity running was increased by 15%. Trial time: E: 8,7 ± 1,8 min P: 7,6 ± 1,5 min (*p* < 0,05)
[17]	Healthy, physically active men; *n* = 15	Beetroot juice	4,4 mg or 0.07 mmol nitrate/kg body WT/day	6 days	Increased exercise test simulating a 5000-m altitude	Short-term dietary nitrate supplementation improves arterial and muscle oxygenation status but not cerebral oxygenation status during exercise in severe hypoxia. Trial duration: E: 597 ± 22 s P: 568 ± 23 s (*p* < 0,05)
[11]	Healthy, recreationally active men; *n* = 10	70, 140, or 280 ml of beetroot juice or placebo (PL) 70, 140, or 280 ml	~ 4.2, ~ 8.4, or ~ 16.8 mmol nitrates or placebo containing ~ 0.04, ~ 0.08, or ~ 0.12 mmol nitrates	4–5 weeks	Two, 5-min bouts of moderate-intensity exercise and one bout of severe-intensity exercise that was continued until task failure as a measure of exercise tolerance, 2.5 h post-ingestion of beetroot juice or placebo.	8.4 and 16.8 mmol of nitrates significantly improved the time-to-task failure by 14 and 12%, respectively, during severe-intensity exercise. End-exercise VO_2_ during moderate-intensity exercise was reduced significantly following the ingestion of 280 ml of BR (*p* < 0.05).
[23]	Healthy, physically active men; *n* = 16	500 ml beetroot juice	5 mmol	1.5 h before physical load	A continuous cycle exercise test involving 20-min stages at 50 and 70% VO_2_max and a final stage at 90% VO_2_ max until volitional exhaustion.	Dietary nitrate reduced VO_2_max by 15,63% and increased the time-to-exhaustion by 16%. Trial duration: E: 185 ± 122 s P: 160 ± 109 s (*p* < 0,05)
[25]	Healthy, recreationally active participants *n* = 15 (males = 8, females = 7)	1000 mg red spinach extract powder (RSE) or placebo (PBO) (maltodextrin) in gelatin capsules	~ 90 mg	One single dose	At one occasion 65–75-min post-RSE/PBO ingestion, venipuncture was performed (PRE), after which graded exercise test - the Bruce protocol - was performed.	Significantly increased plasma NO_3_-. A large effect on the ventilatory threshold compared to the placebo (+ 0.12 ± 0.14 L/min) with the ventilatory threshold occurred at a significantly higher relative VO_2_ (+ 3.6 ± 5.2%, *p* < 0.05).

The mechanisms by which NO_3_ supplementation might influence oxygen uptake and utilization could include a reduction of NO_3_ to NO, directly influencing mitochondrial efficiency, vascular tone and/or tissue oxygenation. However, the evidence for these mechanisms depends on the measurement of NO_2_ in the plasma [9].

Amaranth is a leafy vegetable, and its leaves and grains contain large amounts of NO_3_ as well as other nutrients [26]. The NO_3_ content of amaranth may range from 965 to 4259 mg/kg [1] or from 1800 to 9200 mg/kg [27]. There is scientific evidence suggesting that nitrate-rich spinach can augment nitric oxide levels, enhance endothelial function, and lower blood pressure acutely [13]. Moore and co-authors [25] reported that acute ingestion of 1000 mg of an amaranth extract substantially increased only the plasma NO_3_ level and not the NO_2_ level. Subramanian and Gupta [28] reported that an acute 2000 mg dose of amaranth extract, delivering ~ 180 mg (~ 2.9 mmol) of NO_3_, increased plasma NO_3_ and NO_2_. This increase is similar to, or exceeds that observed with acute ingestion of relatively higher NO_3_ doses from beetroot juice [11, 18].

There were some limitations to our study. We did not measure the plasma contents of NO_3_ or NO_2_; thus, based on the results of other studies, we can only assume that it increased after 6 days of amaranth consumption. Further studies are needed to measure acute and long-term changes in plasma NO_3_ and NO_2_ after the consumption of given amaranth supplement. Secondly, the number of study participants was rather small, so the statistical power was moderate (49.9% for the absolute VO2 max in the experimental group). We did not utilize a crossover type of experiment design in this particular study which is a particularly useful option in evaluating the safe and effective use of tested substances when participants switch from one substance to another. It has both advantages and disadvantages as compared to parallel study. Usually the order effect and carry-over effects are discussed among limitations. In our case the study involved repeated graded exercise tests so often testing might have some training effect. The washout period of ingesting dietary nitrates is unknown, so the experiment could be rather long with increased influences of different covariates. In this case a randomized double-blinded was used as a way of carrying out an experiment in an attempt to minimize subjective biases on the part of the experimenter and on the part of the participant.

## Conclusion

Long-term (6 days) supplementation with dietary NO_3_ from amaranth may improve aerobic capacity during ICE in young physically active male persons. It can be recommended as the nutritional supplement during last week of preparation for competition in endurance events.

## Data Availability

The data that support the findings of this study are available on request from the corresponding Tomas Liubertas, T.L. The data are not publicly available due to General Data Protection Regulation (GRDP) of European Union (restrictions containing information that could compromise the privacy of research participants);

## Abbreviations

ICE: Increased cycling exercise
OB: Oat bar
VT1: First ventilatory threshold
VT2: Second ventilatory threshold
1 T: Test Nr. 1 executed at day 1 of the study
2 T: Test Nr. 2 executed at day 4 of the study
3 T: Test Nr. 3 executed at day 9 of the study
HR: Heart rate
RER: Respiratory exchange ratio
La: Blood lactate concentration
SD: Standard deviation
NO_3_^−^: Nitrate
NO: Nitric oxide
W: Watts
VO_2_max: The maximum rate of oxygen consumption measured during incremental exercise

## References

[1] Lidder S, Webb AJ (2013). Vascular effects of dietary nitrate (as found in green leafy vegetables and beetroot) via the nitrate-nitrite-nitric oxide pathway. Br J Clin Pharmacol.

[2] Santamaria P (2006). Nitrate in vegetables: toxicity, content, intake and EC regulation. J Sci Food Agric.

[3] Hord NG, Tang Y, Bryan NS (2009). Food sources of nitrates and nitrites: the physiologic context for potential health benefits. Am J Clin Nutr.

[4] Stanaway L, Rutherfurd-Markwick K, Page R, Ali A (2017). Performance and health benefits of dietary nitrate supplementation in older adults: a systematic review. Nutrients..

[5] Totzeck M, Hendgen-Cotta UB, Luedike P, Berenbrink M, Klare JP, Steinhoff H-J (2012). Nitrite regulates hypoxic vasodilation via myoglobin-dependent nitric oxide generation. Circulation.

[6] Dreißigacker U, Wendt M, Wittke T, Tsikas D, Maassen N (2010). Positive correlation between plasma nitrite and performance during high-intensive exercise but not oxidative stress in healthy men. Nitric Oxide.

[7] Lundberg JO, Carlstrom M, Larsen FJ, Weitzberg E (2011). Roles of dietary inorganic nitrate in cardiovascular health and disease. Cardiovasc Res.

[8] Larsen FJ, Schiffer TA, Borniquel S, Kent Sahlin K, Ekblom B, Lundberg JO, Weitzberg E (2011). Dietary inorganic nitrate improves mitochondrial efficiency in humans. Cell Metab.

[9] Larsen FJ, Schiffer TA, Weitzberg E, Lundberg JO (2012). Regulation of mitochondrial function and energetics by reactive nitrogen oxides. Free Radic Biol Med.

[10] Bailey SJ, Fulford J, Vanhatalo A, Winyard PG, Blackwell JR, DiMenna FJ, Wilkerson DP, Benjami N, Jones AM (2010). Dietary nitrate supplementation enhances muscle contractile efficiency during knee-extensor exercise in humans. J Appl Physiol.

[11] Wylie LJ, Kelly J, Bailey SJ, Blackwell JR, Skiba PF, Winyard PG, Jeukendrup AE, Vanhatalo A, Jones AM (2013). Beetroot juice and exercise: pharmacodynamic and dose-response relationships. J Appl Physiol.

[12] Bailey SJ, Winyard P, Vanhatalo A, Blackwell JR, DiMenna FJ, Wilkerson DP, Tarr J, Benjamin N, Jones AM (2009). Dietary nitrate supplementation reduces the O_2_ cost of low-intensity exercise and enhances tolerance to high-intensity exercise in humans. J Appl Physiol.

[13] Bond H, Morton L, Braakhuis AJ (2012). Dietary nitrate supplementation improves rowing performance in well-trained rowers. Int J Sport Nutr Exerc Metab.

[14] Cermak NM, Gibala MJ, van Loon LJ (2012). Nitrate supplementation's improvement of 10-km time-trial performance in trained cyclists. Int J Sport Nutr Exerc Metab.

[15] Lansley KE, Winyard PG, Bailey SJ, Vanhatalo A, Wilkerson DP, Blackwell JR, Gilchrist M, Benjamin N (2011). Acute dietary nitrate supplementation improves cycling time trial performance. Med Sci Sports Exerc.

[16] Lansley KE, Winyard PG, Fulford J, Vanhatalo A, Bailey SJ, Blackwel JR, DiMenna FJ, Gilchrist M, Benjamin N, Jones AM (2011). Dietary nitrate supplementation reduces the O_2_ cost of walking and running: a placebo-controlled study. J Appl Physiol.

[17] Masschelein E, Van Thienen R, Wang X, Van Schepdael A, Thomis M, Hespel P (2012). Dietary nitrate improves muscle but not cerebral oxygenation status during exercise in hypoxia. J Appl Physiol.

[18] Vanhatalo A, Bailey SJ, Blackwell J, DiMenna FJ, Pavey TG, Wilkerson DP, Benjamin N, Winyard PG, Jones AM (2010). Acute and chronic effects of dietary nitrate supplementation on blood pressure and the physiological responses to moderate-intensity and incremental exercise. [randomized controlled trial]. Am J Phys Regul Integr Comp Phys.

[19] Larsen FJ, Weitzberg E, Lundberg JO, Ekblom B (2010). Dietary nitrate reduces maximal oxygen consumption while maintaining work performance in maximal exercise. Free Radic Biol Med.

[20] Murphy M, Eliot K, Heuertz RM, Weiss E (2012). Whole beetroot consumption acutely improves running performance. J Acad Nutr Diet.

[21] Peacock O, Tjonna AE, James P, Wisløff U, Welde B, Böhlke N, Smith A, Stokes K, Cook C, Sandbakk O (2012). Dietary nitrate does not enhance running performance in elite cross-country skiers. Med Sci Sports Exerc.

[22] Wilkerson DP, Hayward GM, Bailey SJ, Vanhatalo A, Blackwell JR, Jones AM (2012). Influence of acute dietary nitrate supplementation on 50 mile time trial performance in well-trained cyclists. Eur J Appl Physiol.

[23] Thompson KG, Turner L, Prichard J (2014). Influence of dietary nitrate supplementation on physiological and cognitive responses to incremental cycle exercise. Respir Physiol Neurobiol.

[24] Wasserman K, Hansen JE, Sue DY, Whipp BJ, Casaburi R (1999). Principles of exercise testing and interpretation.

[25] Moore AN, Haun CT, Kephart WC, Holland AM, Mobley CB, Pascoe DD, Roberts MD, Martin JS (2017). Red spinach extract increases ventilatory threshold during graded exercise testing. Sports.

[26] Fasuyi AO, Akindahunsi AO (2009). Nutritional evaluation of *Amaranthus cruentus* leaf meal based broiler diets supplemented with cellulase/glucanase/xylanase enzymes. Am J Food Technol.

[27] Prakash D, Pal M (1991). Nutritional and antinutritional composition of vegetable and grain amaranth leaves. J Sci Food Agric.

[28] Subramanian D, Gupta S (2016). Pharmacokinetic study of amaranth extract in healthy humans: a randomized trial. Nutrition.

